# Microglia as target for anti-inflammatory approaches to prevent secondary brain injury after subarachnoid hemorrhage (SAH)

**DOI:** 10.1186/s12974-021-02085-3

**Published:** 2021-01-30

**Authors:** Rebecca Heinz, Susan Brandenburg, Melina Nieminen-Kelhä, Irina Kremenetskaia, Philipp Boehm-Sturm, Peter Vajkoczy, Ulf C. Schneider

**Affiliations:** 1 Experimental Neurosurgery, Charité – Universitätsmedizin Berlin, corporate member of Freie Universität Berlin, Humboldt-Universität zu Berlin, and Berlin Institute of Health, Berlin, Germany; 2 Department of Experimental Neurology and Center for Stroke Research, Charité – Universitätsmedizin Berlin, Berlin, Germany; 3 NeuroCure Cluster of Excellence and Charité Core Facility 7T Experimental MRIs, Charité – Universitätsmedizin Berlin, Berlin, Germany; 4 Department of Neurosurgery, Charité – Universitätsmedizin Berlin, 10117 Berlin, Germany

**Keywords:** Subarachnoid hemorrhage, Inflammation, Microglia, Secondary brain injury, Inflammatory preconditioning, CSF1-Receptor

## Abstract

**Background:**

Microglia-driven cerebral spreading inflammation is a key contributor to secondary brain injury after SAH. Genetic depletion or deactivation of microglia has been shown to ameliorate neuronal cell death. Therefore, clinically feasible anti-inflammatory approaches counteracting microglia accumulation or activation are interesting targets for SAH treatment. Here, we tested two different methods of interference with microglia-driven cerebral inflammation in a murine SAH model: (i) inflammatory preconditioning and (ii) pharmacological deactivation.

**Methods:**

7T-MRI-controlled SAH was induced by endovascular perforation in four groups of C57Bl/6 mice: (i) Sham-operation, (ii) SAH naïve, (iii) SAH followed by inflammatory preconditioning (LPS intraperitoneally), and (iv) SAH followed by pharmacological microglia deactivation (colony-stimulating factor-1 receptor-antagonist PLX3397 intraperitoneally). Microglia accumulation and neuronal cell death (immuno-fluorescence), as well as activation status (RT-PCR for inflammation-associated molecules from isolated microglia) were recorded at day 4 and 14. Toll-like receptor4 (TLR4) status was analyzed using FACS.

**Results:**

Following SAH, significant cerebral spreading inflammation occurred. Microglia accumulation and pro-inflammatory gene expression were accompanied by neuronal cell death with a maximum on day 14 after SAH. Inflammatory preconditioning as well as PLX3397-treatment resulted in significantly reduced microglia accumulation and activation as well as neuronal cell death. TLR4 surface expression in preconditioned animals was diminished as a sign for receptor activation and internalization.

**Conclusions:**

Microglia-driven cerebral spreading inflammation following SAH contributes to secondary brain injury. Two microglia-focused treatment strategies, (i) inflammatory preconditioning with LPS and (ii) pharmacological deactivation with PLX3397, led to significant reduction of neuronal cell death. Increased internalization of inflammation-driving TLR4 after preconditioning leaves less receptor molecules on the cell surface, providing a probable explanation for significantly reduced microglia activation. Our findings support microglia-focused treatment strategies to overcome secondary brain injury after SAH. Delayed inflammation onset provides a valuable clinical window of opportunity.

**Supplementary Information:**

The online version contains supplementary material available at 10.1186/s12974-021-02085-3.

## Introduction

Aneurysmal subarachnoid hemorrhage (SAH) leads to devastating outcomes, resulting in severe neurological deficits for survivors [1].

Many different factors contributing to brain injury after SAH have been identified for the early and for the late phase. Early brain injury occurs with the initial bleeding, leading to rise of intracranial pressure, disturbance of the blood-brain barrier, cerebral edema, decrease of cerebral perfusion, and early ischemic brain injury with subsequent neuronal cell death [2, 3]. In contrast, secondary brain injury describes a number of pathologies that occur in the later course of SAH, leading to additional brain damage like cortical spreading depolarization, cerebral vasospasm, or post hemorrhagic hydrocephalus [1, 4, 5]. Immune reactions following SAH contribute significantly to secondary brain injury [6–8]. Inflammatory cell invasion and accumulation have been described in a variety of other pathologies like Alzheimer’s disease, ischemic stroke, or traumatic brain injury with detrimental effects [9–11]. Cerebral spreading inflammation after SAH has been described previously by our group and others. Microglia accumulate within the brain tissue between day 4 and 14 after SAH and inflict additional neuronal cell death [6, 12, 13]. The time interval before initiation of secondary brain injury gives rise for potential treatment options.

Microglia, the brain’s innate immune system, originating from the yolk sac [14] get activated upon interaction with pathogens. Once activated, they act through pathogen presentation, production of cytokines/chemokines, and phagocytosis [14]. Microglia activation contributes to neuronal cell death after SAH [6]. An important downstream pathway is Toll-like-receptor 4 (TLR4) [15–17]. TLR4 is a common pattern recognition receptor on the surface of immune cells like microglia. Upon blood-appearance in the subarachnoid space, it recognizes danger-associated molecular patterns like blood components and cell debris and initiates inflammation via intracellular MyD88 and NFκB [18–20].

Considering this cascade of inflammation-driven secondary brain injury, therapeutic strategies targeting microglia activation seem promising. In this study, we describe two different ways of altering microglia activation to overcome neuronal cell death: (a) inflammatory preconditioning and (b) pharmacological deactivation of microglia using colony-stimulating factor 1 receptor (CSF1-R) inhibitor.

Preconditioning is the confrontation of a system with a mild stimulus of the same kind as a subsequently expected noxa with the idea of reprogramming the system’s reaction to that noxa. The commonly best-described type of preconditioning is hypoxic preconditioning, e.g., in cardiac or cerebral ischemia [21]. Mild activation of microglia by low-dose exposure to lipopolysaccharides (LPS) prior to subarachnoid hemorrhage is supposed to reprogram inflammatory signaling after the bleeding. Here, TLR4-signaling and its possible alteration via preconditioning might play an important role [22, 23]. Inflammatory preconditioning has also been investigated in contexts of inflammation after stroke or spinal cord injury where neuroprotective effects have been shown [24–26].

Our second approach to prevent secondary inflammation-driven brain injury is pharmacological deactivation of microglia using a selective CSF1-R/c-kit inhibitor, which acts as a tyrosine-kinase-inhibitor [27]. Within the central nervous system (CNS), microglia exclusively are CSF1-R-signalling-dependent. Blocking the receptor results in a loss of function of microglia [28–30]. Therefore, this pathway is a selective way to reduce microglia activation.

To determine if inflammatory preconditioning or pharmacological deactivation of microglia lead to a reduction of microglia-induced cerebral spreading inflammation and secondary brain injury respectively, we took advantage of a murine model of experimental SAH.

## Materials and methods

### Animal experiments

All experiments were approved by the local authorities (Landesamt für Gesundheit und Soziales Berlin, Germany, RegNr. G0177/14, G0063/18) and committed in conformity with German law of animal protection and the National Institute of Health guidelines for care and use of laboratory animals.

C57Bl/6 wild-type mice (Charles River Laboratories, 12–14 weeks old, body weight app. 21–24 g) were kept at the Forschungszentrum für Experimentelle Medizin, Charité-Universitätsmedizin Berlin.

### Animal model of experimental SAH

A filament perforation model was used for experimental SAH as described elsewhere [31].

Sham-operated animals underwent the same procedure (insertion and advancement of the filament within the internal carotid), but without vessel puncture.

**Fig. 1:**
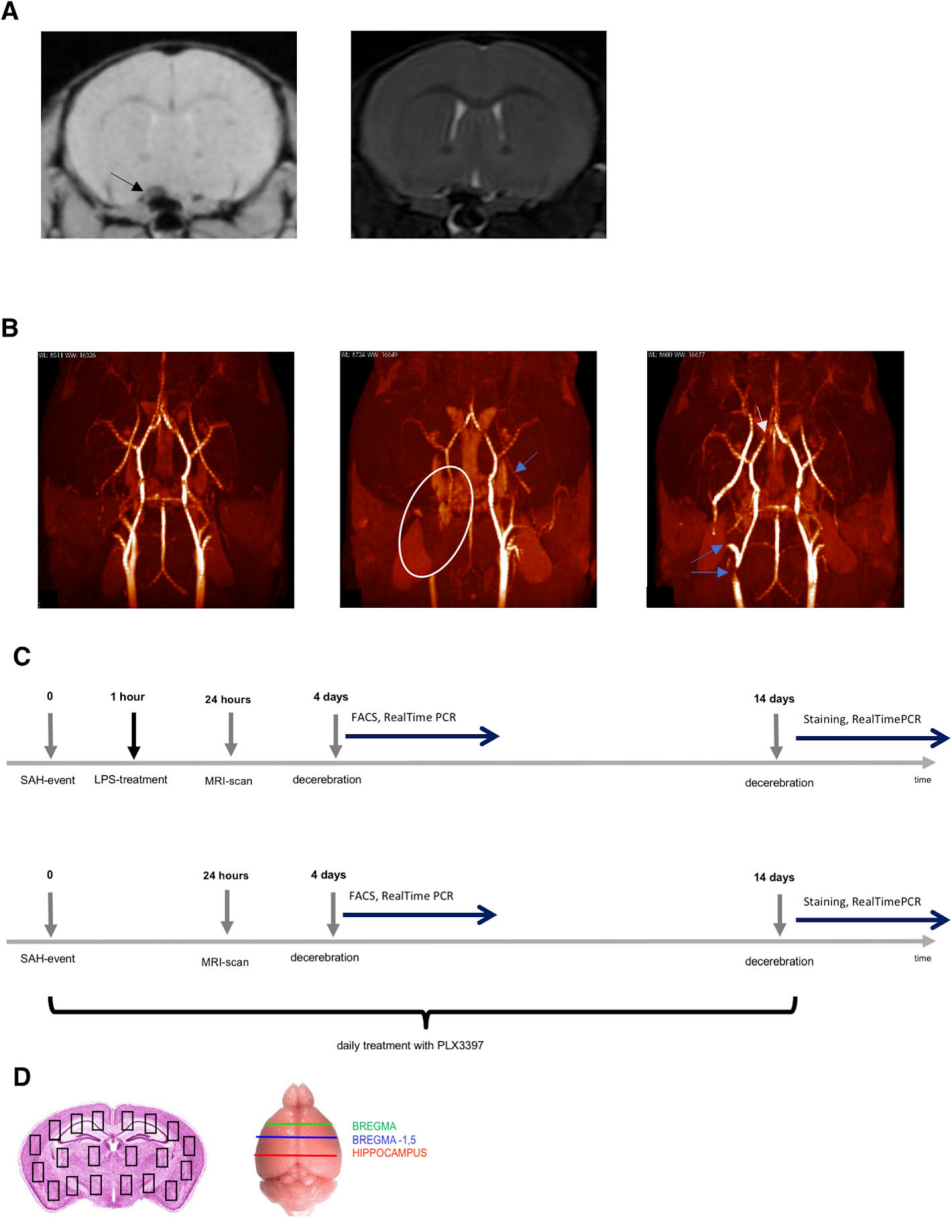
MRI-scans and experimental protocols after SAH. **a** T2*-weighted sequence (24 h), Bregma + 0.5 mm, arrow pointing at SAH, T2-weighted sequence (24 h), ruling out SAH-associated complications like intraparenchymal bleeding or ischemia. **b** Illustrative image of time-of-flight (TOF)-MR-angiography (maximum intensity projection) performed before (left), right after (middle), and in the course of SAH (right). Regular cerebrovascular perfusion was seen before the bleeding. Directly after the bleeding, cerebrovascular structures seemed impaired (arrows), possibly due to a rise of the intracranial pressure and a breakdown of the cerebral perfusion. Cerebrovascular perfusion was restored later (arrows, measurement 3 days after the bleeding). **c** Timeline of experimental protocol for inflammatory preconditioning and pharmacological deactivation using PLX3397 are displayed, indicating the timepoints of interventions and diagnostics as well as treatment duration. **d** Illustration of the 3 representative levels for immunofluorescence analysis (right) and distribution of the representative HPFs for microglia and neuronal cell count (left)

All animals underwent MRI-scans 24 h after SAH using a 7-Tesla rodent scanner (Pharmascan 70/16, Bruker, Ettlingen, Germany) equipped with a 20-mm-diameter transmit/receive volume coil (RAPID Biomedical, Rimpar, Germany). T2*-imaging was performed to assure occurrence of the bleeding. T2-imaging ruled out ischemia or intraparenchymal bleeding (Fig. 1a). MRA-TOF images showed the puncture site of the filament and illustrated dynamic change of the physiological blood flow conditions during the time course of SAH. This procedure was performed with 3 representative animals directly, 24 h and 72 h after SAH-induction (Fig. 1b). Scan sequence details can be provided on demand. Image processing was performed using OsiriX (Pixmeo, Switzerland).

The experiments comprised 4 groups: Sham-operated animals, untreated SAH-animals, LPS-preconditioned SAH-animals, and CSF1R-inhibitor-treated SAH-animals.

A total of 86 animals were included and divided in these 4 groups: 17 Sham-operated animals (mortality rate: 0%), 20 untreated SAH-animals (mortality rate: 10%), 24 preconditioned SAH-animals (mortality rate: 12.5%), and 23 CSF1R-inhibitor treated SAH-animals (mortality rate 8.7%). Group sizes were estimated under statistical supervision, taking readouts from our former studies into account.

Application and dosing were chosen according to prior publications with or without internal modifications. LPS (Sigma-Aldrich) was applied intraperitoneally (0.6 mg/kg) as described before [23, 32]. The preferred application of CSF1R-inhibitor, further referred to as PLX3397 (Plexxikon Inc.), is enteral application with the following variations: mixed into chow, applied via gavage, or mixed into some appealing agent (e.g., corn oil or Nutella®) [33–36]. Parenteral application has been used before, when enteral application seemed inappropriate [37]. Dosing varies substantially between 20 and 50 mg/kg in these studies. Animals after SAH lose weight within the first days. This is why we decided against oral administration, but for intraperitoneal injection. The dose was 30 mg/kg, according to internal communications in our lab and aiming at the mid-range of the aforementioned published data.

LPS was given 1 h after SAH induction. The term “preconditioning” is considered as LPS-administration before inflammation occurs and not before SAH takes place. PLX3397 was applied daily with first application one hour after SAH induction. After 4 and 14 days, animals were perfused with either ice-cold PBS or 4%PFA under deep anesthesia and decapitated (Fig. 1c).

### Immunofluorescence analyses

For immunofluorescence analysis, brains of 20 mice (5 animals per group) were fixed in 4%PFA for 24 h, dehydrated in 30% saccharose for another 24 h, and cut into 10 μm coronal sections.

Immunofluorescence staining was performed for microglia (Iba-1) and neuronal cell death (NeuN/TUNEL co-staining). In previous experiments, we showed that Iba1-positive cells are microglia exclusively, and not secondarily invaded macrophages/lymphocytes [6]. Slides were mounted and nuclei labeled with DAPI mounting medium in all protocols (Dianova). Primary antibodies were applied at 4 °C overnight. Secondary antibodies were incubated for 2 h at room temperature. Antibodies used included rabbit anti-Iba-1 (1:250 WAKO), mouse anti-NeuN (1:200 Millipore), DyLight 488 donkey anti-rabbit (1:200 Jackson), and FITC donkey anti mouse (1:100 Jackson). Neuronal cell death was detected by TUNEL (ApopTag© Red In Situ, Millipore), according to manufacturer’s protocol.

For quantitative analysis, whole sections were mounted and analyzed under a fluorescence microscope (Zeiss, Axio Observer Z1, Carl Zeiss GmbH, Munich, Germany) equipped with a digital camera (AxioCam MRc). Immunofluorescence sections were divided into 20 standardized high-power fields (HPFs, allowing for total cell counts per brain section on three different sections of the brain (bregma + 0 mm, bregma + 1.5 mm, bregma + 3 mm). Mean values and standard deviation were calculated per animal from 20 HPF/section (× 3 sections) (Fig. 1d).

### Isolation of microglia from murine brains

In a separate set of experiments, murine brains from a total of 33 animals were harvested on day 4 (*n* = 17) or day 14 (*n* = 16) after the bleeding. Group sizes were 3 sham, 4 SAH, 5 LPS-preconditioned, and 5 PLX3397-treated on day 4 and 3 sham, 3 SAH, 5 LPS-preconditioned, and 5 PLX3397-treated on day 14, respectively. The brains were brought into suspension, taking advantage of the MACS^©^ Neural Tissue Dissociation Kit, according to manufacturer’s recommendation, using gentleMACS Octo Dissociator. To this suspension, a magnetic microbead-labeled CD11b-antibody (all Miltenyi Biotec) was added. After incubation at 4° for 15 min, the respective cell pools were added to a pre-cooled column placed within a magnet. This procedure was repeated twice to achieve higher purity. The purity of this CD11b-positive microglia cell fraction was evaluated by FACS, using antibodies against CD11b and CD45 (BD Bioscience, FITC-coupled, M1/70, APC-coupled 30-F11). DAPI (1:100, Sigma Aldrich) was added to the cell suspension to exclude dead cells before samples were measured using FACS (BD FACS Canto II, BD Bioscience, Germany), getting an overall purity of 90%.

### Molecular analysis of the isolated microglia

RNA extraction was performed from the isolated microglia cell fraction, using RNA Extraction Kit (NucleoSpin^©^ RNA II, Fisher Scientific, UK). The amount of RNA was evaluated at 260 nm filter range. From RNA, cDNA was reversely transcribed using PrimeScript™RT Reagent Kit with gDNA Eraser (TaKaRa Bio Inc.), according to manufacturer’s recommendation.

Quantitative real-time PCR (RTq-PCR) was then performed for the inflammatory cytokines TNFα, IL1β and IL6, the anti-inflammatory cytokines IL4, IL10, and TGFβ, and the receptor TLR4 using SYBR® Premix Ex Taq (TaKaRa Bio Inc.). All primers were produced by TIB MOLBIOL (Berlin, Germany, primer sequences provided in suppl table). Results are displayed as relative quantity normalized to sham values (RQ, delta-delta CT model). Hence, statistical comparison to sham values was considered mathematically incorrect.

### Staining for FACS analysis

For FACS analysis, the brains of 24 animals were used (*n* = 6 per group). CD115 and TLR4 staining was realized using the following antibodies: FITC anti-CD11b (M1/70, Bio-Legend), PerCP anti-CD45 (30-F11, BD Biosciences), PE anti-CD115 (T38-320, BD Biosciences), and APC anti-TLR4 (SA15-21, Bio-Legend). Here, CD11b^+^ CD45^+^ cells were gated and afterwards the percentage of TLR4-positive and CD115-positive cells was calculated. DAPI (1:100, Sigma Aldrich) was added to the cell suspension to exclude dead cells.

All samples were measured using FACS and evaluated with FlowJo software (Tree Star).

### Statistical analysis

Statistical analyses were performed using GraphPad PRISM (GraphPad Software, version 8.2). All data were analyzed by one-way ANOVA with Bonferroni correction for multiple comparisons to detect statistical differences. All cell count values are displayed as means ± standard deviation. Statistical significance was defined at *p* < 0.05, *p < 0.05, **p < 0.01, ***p < 0.001, and ****p < 0.0001.

## Results

### Inflammatory preconditioning and PLX3397-treatment both reduce SAH-dependent microglia accumulation

**Fig. 2:**
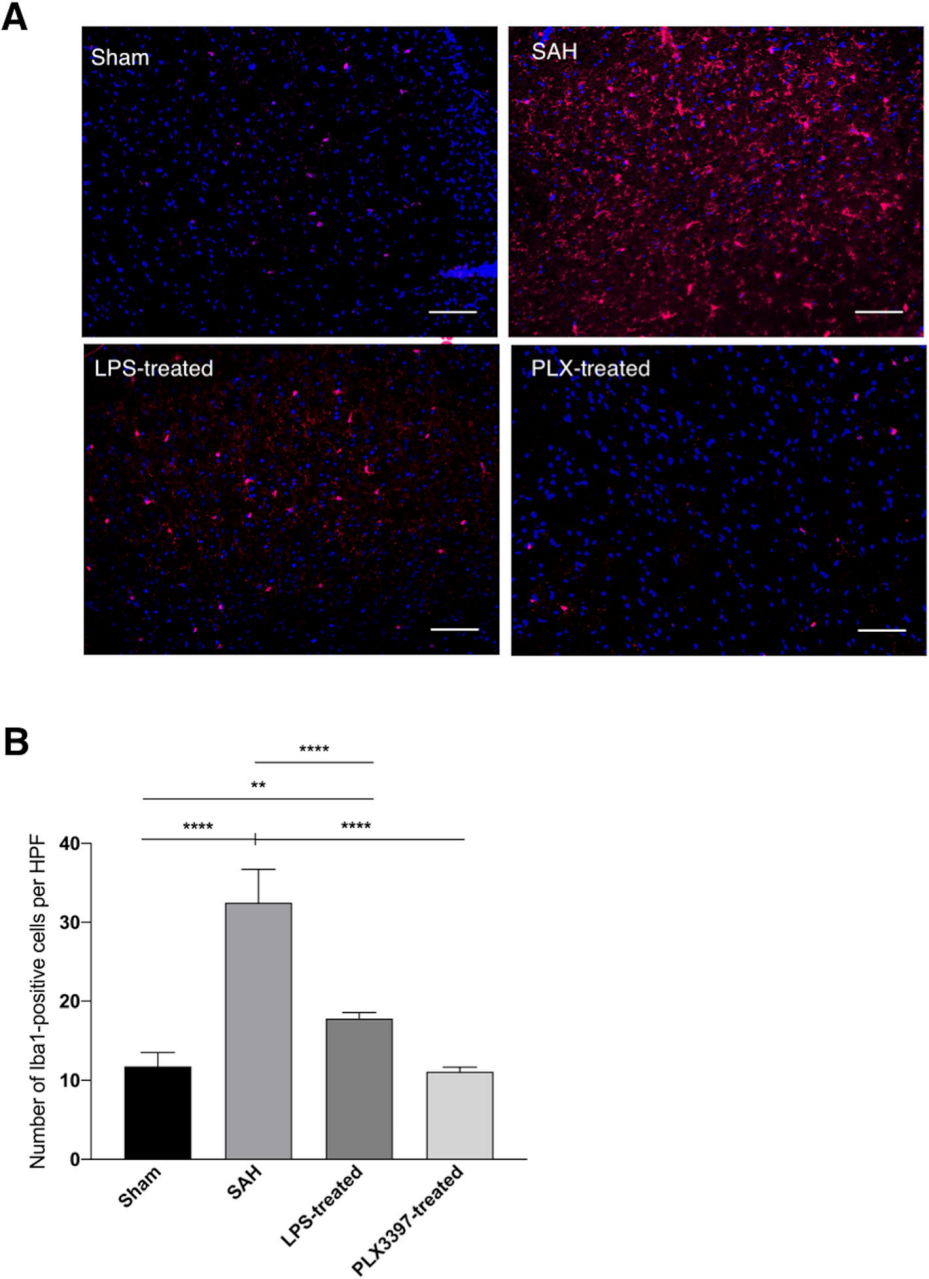
Microglia accumulation showing cerebral spreading inflammation after SAH (day 14). **a** Representative immunofluorescence staining of microglia (Iba1) within the brain parenchyma. While in Sham animals only minimal microglia-accumulation was seen (11.157 ± 1.538 cells/HPF, upper left), a significant increase was detected after SAH (32.79 ± 4.77 cells/HPF, upper right, *p* < 0.0001). LPS preconditioning led to significantly diminished microglia cell numbers in comparison to SAH without treatment, but still showed more microglia accumulation in comparison to sham animals (17.43 ± 2.42 cells/HPF, lower left, *p* < 0.0001 vs. SAH, *p* < 0.01 vs. Sham), while in PLX3397-treated animals nearly sham-levels were reached (11.16 ± 1.54 cells/HPF, lower right, *p* < 0.0001 vs. SAH without treatment). Scale bar = 100 μm. **b** Mean cell counts ± standard deviations displayed in bar diagram showing the significant differences in microglia accumulation between the four treatment groups. *n* = 5 per group, ANOVA: **p < 0.01; ****p < 0.0001

As in our previous experiments, SAH led to significant microglia accumulation within the brain parenchyma until day 14 after the bleeding, compared to sham-operated animals (SAH: 32.79 ± 4.77 vs. Sham 11.157 ± 1.538 cells/HPF, *p* < 0.0001). Our current data show that both treatments, inflammatory preconditioning (17.43 ± 2.42 cells/HPF), as well as PLX3397-treatment (11.16 ± 1.54 cells/HPF) significantly reduced microglia accumulation within the time course of SAH compared to untreated SAH animals (both *p* < 0.0001, Fig. 2).

### Inflammatory preconditioning and PLX3397-treatment both reduce SAH-driven neuronal cell death

**Fig. 3:**
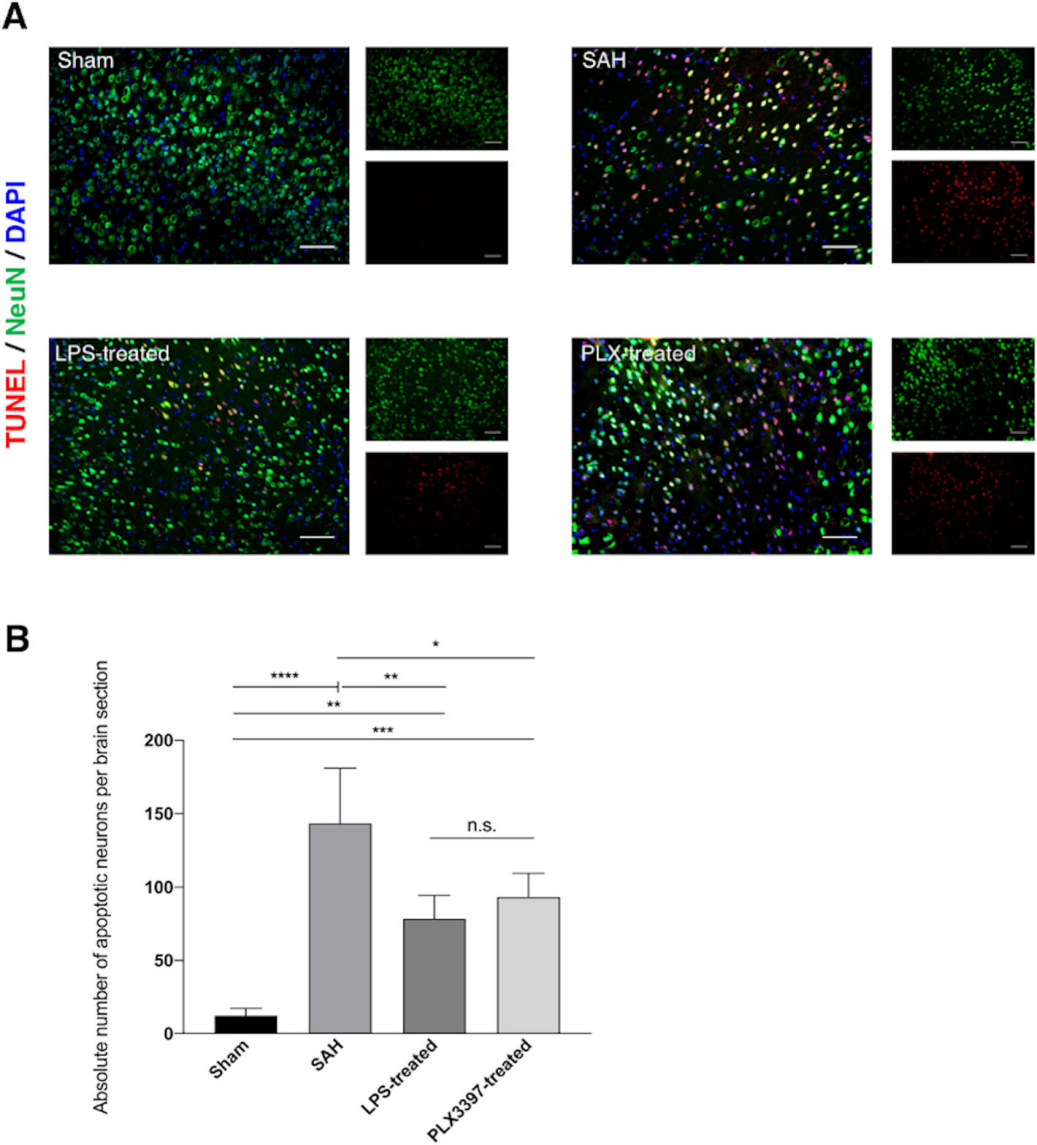
Neuronal cell death after SAH (day 14). **a** Representative immunofluorescence staining of neuronal nuclei (NeuN, green) and cell death (TUNEL, red). Images of each group are displayed as single staining results for NeuN and TUNEL in a smaller scale and a fused image in large scale displaying both cells (green or red) and co-staining (yellow), as well as nuclei (DAPI, blue). Scale bar = 100 μm. In Sham animals (upper left), dying neurons could only scarcely be detected (12.2 ± 4.453 dying neurons/brain section). SAH induced significant neuronal cell death, as documented before (141,821 ± 60,866 dying neurons/brain section, upper right, *p* < 0.0001 vs. Sham). Preconditioning by LPS successfully prevented neuronal cell death (78.4 ± 28.625 dying neurons/brain section, lower left, *p* < 0.01 vs. SAH), as did PLX3397 treatment (92.5 ± 37.267 dying neurons/brain section, lower right, p<0.05 vs. SAH). Still, more neuronal cell death was documented in both treatment groups, when compared to sham animals (*p* < 0.01 LPS vs. Sham, *p* < 0.001 PLX3397 vs. Sham). **b** Mean cells counts and standard deviations displayed in bar diagram showing the significant differences in neuronal cell death between the four treatment groups as described above. *n* = 5 per group, ANOVA: *p < 0.05, **p < 0.01, ***p < 0.001, ****p < 0.0001

Microglia accumulation within the brain tissue has previously been found to initiate neuronal cell death. In line with these previous results, we confirmed a significantly increased amount of dying neurons in animals after SAH as compared to sham-operated animals (dying neurons/brain section SAH: 141,821 ± 60,866 vs. Sham: 12.2 ± 4.453, *p* < 0.0001). In animals in both treatment groups, significantly less neuronal cell death was detected (dying neurons/brain section LPS preconditioning: 78.4 ± 28.625, *p* < 0.01 vs. SAH, PLX3397 treatment: 92.5 ± 37.267, *p* < 0.05 vs. SAH). However, we saw no significant difference between the two treatment groups (Fig. 3).

### Inflammatory preconditioning and PLX3397-treatment both modify pro- and anti-inflammatory gene-expression of microglia after SAH

We analyzed the pro- and anti-inflammatory gene-expression profiles of microglia isolated from the brains of our four treatment groups.

**Fig. 4:**
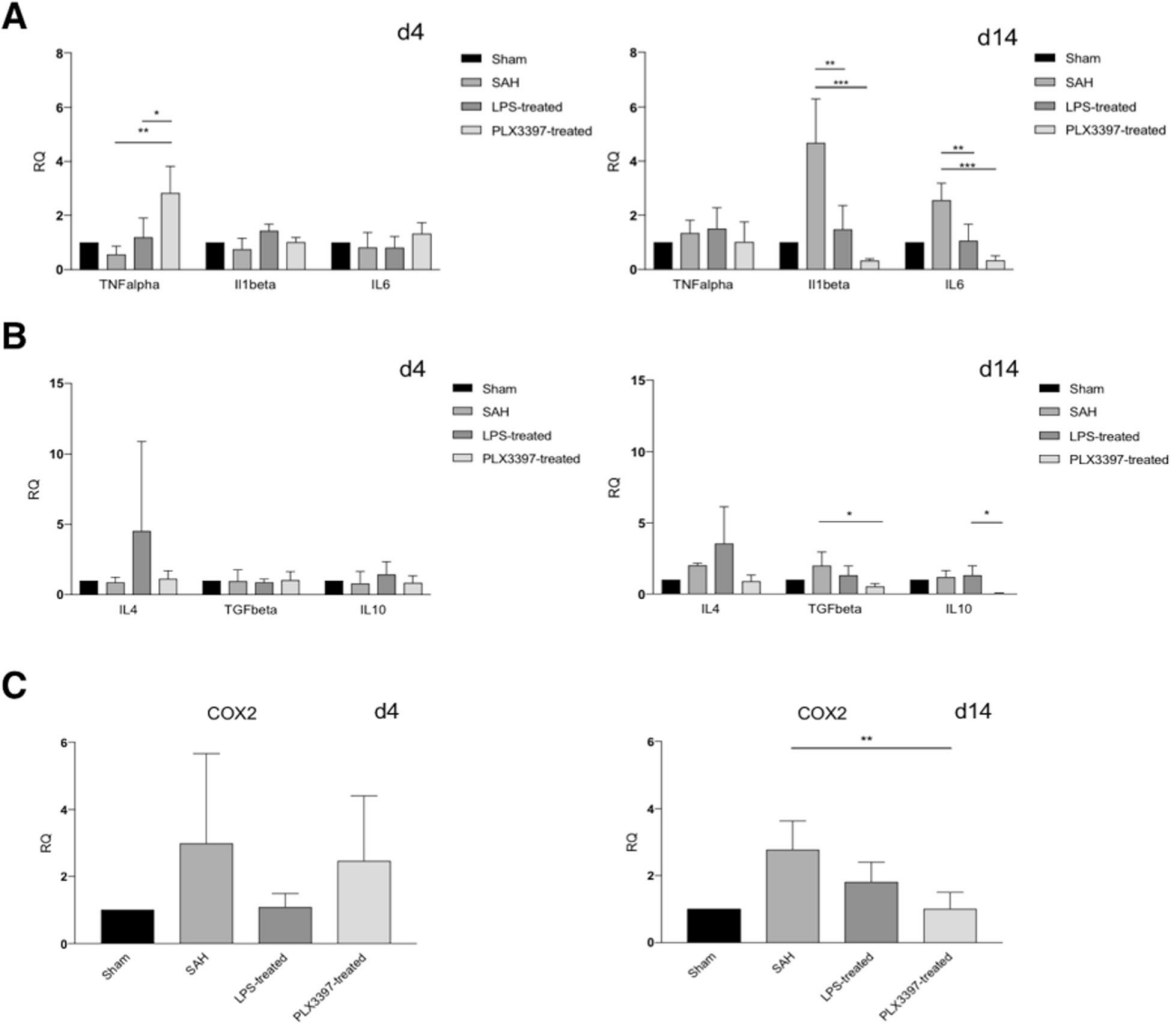
Pro- and anti-inflammatory gene-expression profiles of isolated microglia by qPCR. Early after the bleeding (day 4), individual changes in pro (**a**)- and anti-inflammatory (**b**) gene expression levels were seen. **a** Only TNFα levels after PLX3397-treatment were significantly elevated (3-fold compared to Sham, *p* < 0.01 and 2-fold compared to LPS treatment *p* < 0.05) early after the bleeding. Fourteen days after the bleeding, a significant rise of pro-inflammatory cytokine-levels was documented in SAH-animals (IL1β: 4.7-fold compared to Sham, IL6: 2,3-fold compared to sham). Inflammatory preconditioning as well as PLX3397-treatment led to significant amelioration of the gene expression near sham values (IL1β/IL6: LPS pretreatment vs. SAH *p* < 0.01, PLX3397 treatment vs. SAH *p* < 0.001). **b** For anti-inflammatory cytokines, no significant changes were seen early after the bleeding (day 4). On day 14 after the bleeding, PLX3397-treatment led to a significant loss of gene-expression of TGFβ when compared to SAH and of IL10 when compared to LPS preconditioning (both *p* < 0.05). At both time points, 4-fold elevation of IL4 after LPS preconditioning did not reach significance levels due to high standard deviation. **c** In line with the pro-inflammatory cytokines, levels of the pro-inflammatory enzyme COX2 showed only slight changes on day 4 (n.s.). On day 14, a significant reduction was seen in PLX3397-treated animals only when compared to SAH (*p* < 0.01), *n* = 3–5 per group, ANOVA: *p < 0.05; **p < 0.01; ***p < 0.001, normalized to Sham-group (RQ = relative quantity, statistical comparison to sham group not given due to normalization for delta-delta CT model)

In pro-inflammatory genes, at day 4, no significant difference in the untreated SAH-group as well as in the preconditioned group was detected in comparison to sham-operated animals, indicating no significant inflammatory activation of microglia in these groups. However, the PLX3397-treated group showed a significant increase of TNFα gene-expression at this early time point as compared to the other groups (PLX3397 treatment vs. SAH *p* < 0.01, PLX3397 treatment vs. LPS preconditioning *p* < 0.05, Fig. 4a, left).

At day 14, an increase of IL6 and IL1β was seen in the SAH-group, as compared to sham-operated animals, but not in TNFα anymore. In animals after inflammatory preconditioning, gene-expression of IL6 and IL1β was significantly lower than in SAH animals without treatment and comparable to Sham-level. In PLX3397-treated animals, even lower gene expression levels for these two molecules were seen (IL1β and IL6: LPS preconditioning vs. SAH *p* < 0.01, PLX3397 treatment vs. SAH *p* < 0.001, Fig. 4a, right).

Concerning anti-inflammatory genes (IL4, IL10, and TGFβ), a slight but not significant upregulation of IL4 was seen in the LPS preconditioned group at day 4 and 14 (n.s.).

Furthermore, at day 14 in PLX3397-treated animals, significantly lower levels of anti-inflammatory cytokines were found, while no significant changes were seen after LPS preconditioning (PLX3397 treatment vs. SAH: *p* < 0.05, LPS preconditioning vs. SAH n.s., Fig. 4b, right). PLX3397 treatment thus seems to initiate a more complex downstream cascade than LPS preconditioning, allowing for an early increase in TNFα and a later increase of the two other inflammatory cytokines, while showing inhibitory potential in the expression of anti-inflammatory cytokines at day 14 after the bleeding.

COX2 upregulation as consequence of high levels of inflammatory cytokines (IL6, IL1β) has been described before [38], following a similar time course. At day 4, we saw no significant increase in any of our groups, whereas at day 14 an increase of COX2 gene-expression in the SAH group with lower levels in both treatment groups was documented. However, only PLX3397-treatment led to significant changes (LPS preconditioning vs. SAH: n.s., PLX3397 treatment vs. SAH: *p* < 0.01, Fig. 4c).

### Inflammatory preconditioning and PLX3397-treatment both lead to altered TLR4-receptor expression on microglia after SAH

**Fig. 5:**
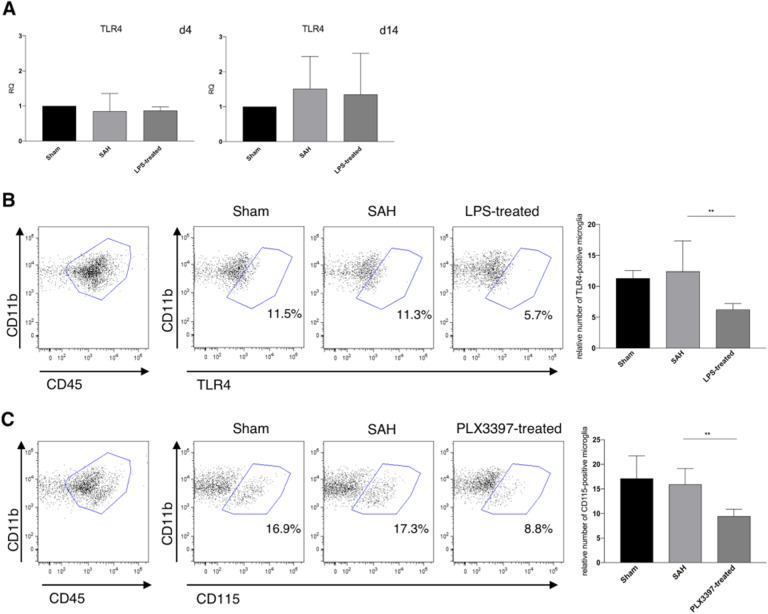
Receptor surface expression of TLR4 and CD115. One known mechanism of inflammation is the TLR4 pathway. **a** TLR4 gene expression in isolated microglia was evaluated using qPCR. No significant changes of gene expression levels were seen at days 4 and 14 after the bleeding (n.s.). **b** As internalization of TLR4 is a potential mechanism of LPS preconditioning, the relative amount of TLR4 on the surface of microglia was determined using FACS-staining (d4). While TLR4-levels on microglia of Sham and SAH-animals were comparable (n.s.), in LPS-preconditioned animals significantly lower numbers of microglia presenting TLR4 on their surface were found, supporting the proposed mechanism of TLR4 internalization upon the inflammatory stimulus. (Proportion of microglia presenting TLR4 on their surface: Sham 11.5%, SAH 11.3%, LPS preconditioning 5.7%, *p* < 0.01 vs. both other groups). **c** As a proof of concept for pharmacological microglia deactivation by PLX3397, FACS-staining was performed for microglia positive for CSF1R (CD115). A significant decrease of CD115^+^ microglia was detected after PLX3397 treatment, when compared to both other groups (Proportion of microglia presenting CSF1R on their surface: Sham 16.9%, SAH 17.3%, PLX3397 treatment 8.8%, *p* < 0.01 vs. both other groups). *n* = 6 per group, ANOVA: **p < 0.01

In a next step, we addressed whether there is a regulation in the expression of TLR4. Baseline qPCR measurements of the gene expression of TLR4 in isolated microglia after inflammatory preconditioning did not show any significant difference in any of the groups (Fig. 5a).

As TLR4 is located on the cellular surface, internalization of the receptor after preconditioning with LPS is a supposed mechanism to explain the anti-inflammatory effect of inflammatory preconditioning. Thus, we analyzed the cellular expression of TLR4 using FACS at day 4 after SAH. The results showed a significant decrease of TLR4 surface expression after inflammatory LPS preconditioning, while values were comparable between the other groups (proportion of microglia presenting TLR4 on their surface: Sham 11.5%, SAH 11.3%, LPS preconditioning 5.7%, *p* < 0.01 vs. both other groups, Fig. 5b).

PLX3397 is an inhibitor of CSF1-R (CD115). The survival signaling of CSF1-dependent cells is less effective after PLX3397 treatment. We therefore investigated the density of the CSF1R on microglia using FACS and found that inhibition of the receptor resulted in a decrease of its surface expression as well (proportion of microglia presenting CSF1R on their surface: Sham 16.9%, SAH 17.3%, PLX3397 treatment 8.8%, *p* < 0.01 vs. both other groups, Fig. 5c).

## Discussion

In our current study, we compared two ways to interact with microglia accumulation and activation within the brain tissue after experimental SAH in mice to overcome secondary neuronal cell death.

Both treatment methods significantly altered microglia accumulation and their activation status, respectively, leading to a significant reduction of neuronal cell death that compared favorably to our previous data [6].

Inflammatory cells within the CNS do not only comprise microglia, but also peripheral monocytic cells (e.g., macrophages or lymphocytes) that may secondarily invade the brain from the periphery through an altered or even disrupted blood brain barrier. We have addressed this topic in prior studies. Using a chimeric mouse model, we showed that peripheral cell invasion into the brain did not occur after experimental SAH. Since our current experiments followed the same protocol, we assume that the Iba1-positive cells detected here are brain resident microglia as well and not peripheral monocytic cells [6].

In our current study, we focused on day 14 after the bleeding. This is the time point of maximum intention of inflammation and neuronal injury, as we have determined in earlier studies [6]. Especially the time interval between occurrence of the bleeding and first documented occurrence of cellular inflammation within the brain tissue, associated with neuronal injury respectively, offers a target for treatment or prevention. Unlike in ischemic stroke or intracerebral hemorrhage, where the time interval for treatment comprises only the first few hours, the possible time interval for SAH according to our data is much longer.

In the context of intracerebral hemorrhage, PLX3397 treatment has been investigated before. In that study, a 14-day pre-treatment led to a 90% reduction of microglia and to favorable outcomes, respectively [39]. Yet, any kind of pre-treatment does not resemble a realistic clinical situation. In our experiments, PLX3397-treatment was started 1 h after induction of SAH, resembling a quite realistic possibility of a clinical setting. Still, treatment resulted in significantly (yet not 90%) reduced microglia cell count and less neuronal cell death 14 days later, underlining the favorable window of opportunity for treatment. The fact that PLX3397 treatment needs several days (in some literature up to weeks [28, 40]) for maximum depletion of microglia might exhibit a potential weakness in this “realistic” treatment scenario, as compared to a pre-treatment scenario. Although we have not performed microglia staining at day 4 after the bleeding—as done in our previous studies that showed only minimal accumulation, repetitively—we discuss two possible theories for our documented significant elevation of TNFα gene expression after PLX3397 treatment on day 4. Given the time course of depletion of microglia under PLX3397 treatment, we hypothesize that on day 4 after the bleeding, there is still a sufficient amount of microglia that remains or becomes activated in the early phase after the bleeding to explain this rise in TNFα gene expression which later is ameliorated, when depletion by PLX3397 progresses (day 14). Nevertheless, this finding remains controversial, as neither IL1β nor IL6 gene expression levels are elevated at day 4. A second possible explanation is another (unknown) contributor. Inflammatory gene expression has been measured in isolated microglia (purity > 90%). Within the residual 10%, we might speculate on another potential contributor to cerebral spreading inflammation that has not been addressed by our experiments. Yet, in light of the existing literature on PLX3397 depletion kinetics, we favor the first option and have to state that early administration of PLX3397 substantially ameliorates inflammatory secondary brain injury following eSAH, but due to long-term depletion kinetics harbors potential drawbacks in the early phase.

The concept of inflammatory preconditioning is new. It has been previously evaluated only recently in ischemic stroke [41] or spinal cord injury [26], but not in SAH. Here, we show marked effects on inflammation itself and the consecutive downstream pathways. Regarding these downstream mechanisms, our first hypothesis was a change in microglia phenotype from inflammatory to anti-inflammatory (M1/M2 shift). However, the cytokine expression profile of our microglia did not completely support this hypothesis. Furthermore, established markers or contributing factors for an M1/M2 shift were not evaluated in our study (e.g., mTOR, CX3CR1, or electrophysiological features) [42, 43]. An additional pathway influencing microglia phenotype is an alteration of TLR4 on the cell surface, that is known to significantly contribute to pro- as well as anti-inflammatory changes in ischemia or traumatic CNS injury [44, 45]. In our experiments, preconditioning with LPS leads to a reduced surface expression of TLR4, but unchanged relative quantity in qPCR. Internalization (due to activation) of the receptor is the only suitable explanation for this constellation, although intracellular staining has not been done. The receptor is further not available for signaling when potential danger-associated molecular patterns (DAMPs) like hem, methemoglobin, and other already described ligands are presented in the course of hemorrhage [17, 46, 47]. Given that microglia are phagocytic cells fulfilling their task as edaphic macrophages, the TLR4 pathway is essential for the immune response mediated by microglia [15, 16, 48–50]. Various components of this pathway have already been investigated in anti-inflammatory approaches regarding SAH; antagonizing inflammatory cytokines, especially IL1β or inhibition of the adapter protein MyD88 [51]. Our current results in accordance with these previous results support the significance of the TLR4 pathway in cerebral spreading inflammation after SAH. Influence by preconditioning seems to be rather easy—and rather cheap—in comparison to the previously published molecular approaches, that mostly also target the intracellular compartment.

The systemic—and thus very easy—application of LPS intraperitoneally in our animals leads to a marked CNS effect. While we have not taken further effort in verifying the transfer steps from peripheral administration to cerebral inflammation, the mechanisms seem to be well-established and have been sufficiently described by other groups. In 2005 and 2007, basic research showed for the first time that peripherally administered LPS causes inflammation effects within the brain (also via TNF-alpha) [52, 53]. The first group to describe intracerebral effects of peritoneally applied LPS in SAH setting were Provencio and co-workers, who also speculated on the TLR4-pathway as downstream mechanism [32, 54]. Wang and collaborators also used LPS to initiate cerebral inflammation [55]. With its molecular size of 50–100 kDa, LPS is able to pass the blood-brain barrier (BBB) in minimal amounts only—if properly closed yet [56], disruption or even pre-sensitization of the BBB through SAH might lead to much larger amounts of LPS to enter the brain [57].

The animal model we applied was the intravascular filament perforation model. Although other models exist, that allow more homogeneous sizes of the bleeding or also more severe bleeding situations (like the autologous blood injection model), we appreciate that the perforation model inflicts vascular rupture at the site of the bleeding, therefore contributing to vessel wall shear stress with the subsequent molecular stress mechanisms. In a previous work, we showed the necessity of intravascular inflammation as precursor of intracerebral inflammation and therefore used the perforation model in the current experiments as well [8].

Although it would be interesting—especially in the clinical situation—if systemic administration of CSF1R antagonists elicits effects on the peripheral immune system, we have not investigated this in detail. We had no deaths in the PLX3397 group that were obviously infection-triggered and drop-out rates did not differ significantly from the other SAH groups.

Behavioral or neurocognitive testing would have been favorable to support our structural read-out by functional data. The general lack of neurocognitive testing in mice in this certain model of SAH is most likely due to the rather mild type of bleeding and the narrow threshold for intracranial compensation in mice compared to rats. While in rats, neuro-testing following SAH has been well-established and extensively reported, reliable and reproducible testing methods for this particular model in mice are still lacking. In various testing methods—including extensive catwalk testing, anxiety test, and depression scales, we could not detect any robust significant differences in mice after SAH induced by filament perforation (own data not published). Like in humans, only subtle neurocognitive decline might follow milder hemorrhage patterns. Using the existing testing battery, these are easily overseen in animals.

As a proof of concept for PLX3397-treatment, we investigated the surface expression of CSF1R (CD115), which is inhibited as shown by FACS analysis (Fig. 5c). The current understanding is that CSF1R is a tyrosine-kinase which is inhibited by PLX3397 in its autophosphorylation-ability [27]. In addition, our data show a lower surface expression. Yet it remains unclear if this is just an indirect sign of receptor deactivation, or an additional effect caused by loss of function of deactivated microglia [58].

We acknowledge that a direct comparison of our two models lacks a methodological similarity.

While LPS-preconditioning “pre-sensitizes” the brain’s immune system, PLX3397 treatment effectively inhibits microglia in their function, thus leading to *immuno*-*suppression.* LPS-preconditioning alters microglia function and might therefore even elicit even more beneficial effects than categorical immuno-suppression by PLX3397, especially in light of the prolonged time to depletion of PLX3397, that we discussed above. Also, anti-inflammatory genes were significantly suppressed after PLX3397 treatment, while they remained on baseline levels after LPS preconditioning.

Interestingly, this was not supported by our findings for neuronal cell death. Although the number of dying neurons was higher after PLX3397 treatment than after LPS preconditioning, no statistically significant difference was found (*p* = 0.2).

Nevertheless, both treatment options effectively counter microglia-mediated secondary brain injury following SAH. Although their mechanisms of action do significantly differ, both offer promising clinical potential, through easy application, low costs, and wide availability. Both treatment options directly act through change in microglia activation, which are the shared detrimental agent for neuronal cell injury. We therefore feel that thorough discussion of the differing mechanisms, but common target and purpose, makes comparison of the results of our two treatment options feasible.

## Conclusion

Increasing evidence shows the crucial role of microglia-driven cerebral spreading inflammation in secondary brain injury after SAH. We have shown before that the phenomenon is not restricted to experimental animal models but observed in humans as well [6]. Here, we evaluated two possible ways of countering the inflammatory response, that both prevent secondary neuronal injury. Due to the easy (and cheap) way of application, and the clinically highly valuable window of opportunity for treatment, inflammatory preconditioning as well as microglia-deactivation through a CSF1R-antagonist both have become valuable clinical treatment options and should be further evaluated in clinical trials.

## Supplementary Information

**Additional file 1: Table S1** Primer sequences

## Data Availability

All data generated or analyzed during this study are included in this published article (and its supplementary information files).

## Abbreviations

SAH: Subarachnoid hemorrhage
TLR4: Toll-like receptor 4
CSF1-R: Colony-stimulating factor 1 receptor
LPS: Lipopolysaccharides
CNS: Central nervous system
BBB: Blood-brain barrier
DAMP: Danger-associated molecular pattern
