# Supplementary material for: Microglia as target for anti-inflammatory approaches to prevent secondary brain injury after subarachnoid hemorrhage (SAH)
Source: J Neuroinflammation. 2021 Jan 30;18:36. doi: 10.1186/s12974-021-02085-3 (PMC7847606; doi:10.1186/s12974-021-02085-3)
Supplement: Supplementary file 1 — Additional file 1: Table S1 Primer sequences [file 12974_2021_2085_MOESM1_ESM.pdf]

Table 1
Primer sequences

Gene	Primer sequence
Cox2	Fw 5'-TCATCAGTTTTTCAAGACAGATC-3'
	Rw 5'-ACCTGATATTTCAATTTTCCATCC-3'
Il1β	Fw 5'-GGCTCACTTCATGAGACTTGC-3'
	Rw 5'-AGGTGTAAGGTGCTGATCTGG-3'
Il4	Fw 5'ATTTTGAACGAGGTCACAGGAGAAG-3'
	Rw 5'-ACCTTGGAAGCCCTACAGACGAG-3'
Il6	Fw 5'-GACTGATGCTGGTGACAACC-3'
	Rw 5'-TTCTGCAAGTGCATCATCG-3'
Il10	Fw 5'-CCCTGGGTGAGAAGCTGAAG -3'
	Rw 5'-CACTGCCTTGCTCTTATTTTCACA -3'
Tgfβ	Fw 5'-GCGTCTGCTGAGGCTCAAGT-3'
	Rw 5'-CTCAACCACTGCCGCACAA-3'
Tlr4	Fw 5'-AAGAAACGGCAACTTGGACC-3'
	Rw 5'-GGAAAGGAAGGTGTCAGTGC-3'
Tnfα	Fw 5'-GACAGTGACCTGGACTGTGG-3'
	Rw 5'-TCTGTGAGGAAGGCTGTGC-3'
18S	Fw 5'-AACCCGTTGAACCCCATT-3'
	Rw 5'-CCATCCAATCGGTAGTAGCG-3'
